# AlienTrimmer removes adapter oligonucleotides with high sensitivity in short-insert paired-end reads. Commentary on Turner (2014) Assessment of insert sizes and adapter content in FASTQ data from NexteraXT libraries

**DOI:** 10.3389/fgene.2014.00130

**Published:** 2014-05-13

**Authors:** Alexis Criscuolo, Sylvain Brisse

**Affiliations:** ^1^Institut Pasteur, Microbial Evolutionary GenomicsParis, France; ^2^Institut National de la Recherche Agronomique, UR1077, Unité Mathématique, Informatique et GénomeJouy-en-Josas, France; ^3^Centre National de la Recherche Scientifique, UMR3525Paris, France

**Keywords:** Alien Trimmer, cutadapt, paired-end reads, Nextera®, transposase sequence, index, primer, insert

In a recent work, Turner (2014) compared the performances of two bioinformatics programs, cutadapt (Marcel, 2011) and AlienTrimmer (Criscuolo and Brisse, 2013), to trim off exogenous oligonucleotides from short-insert paired-end reads. Turner (2014) suggested that AlienTrimmer performed with very low sensitivity. Here we show that this reported lack of performance was due to inappropriate use of AlienTrimmer. Indeed, when all relevant oligonucleotide sequences to be trimmed off are specified, AlienTrimmer performs faster than cutadapt and with equally satisfactory sensitivity.

The bioinformatics protocol by Turner (2014) allows short-insert paired-end reads as well as library preparation oligonucleotides occurring within such reads to be identified. These exogenous oligonucleotide sequences need to be removed from such reads as their presence may affect negatively downstream analyses such as *de novo* assembly or variant detection by mapping approaches (e.g., Criscuolo and Brisse, 2013; Bolger et al., 2014). To illustrate this protocol, Turner (2014) generated paired-end reads of length 250 base pairs (bps) from *Escherichia coli* with 96 separate libraries prepared using the standard dual index Nextera® XT transposon protocol. Figure 1A shows an example of typical paired reads with short insert size from these data. As underlined by Turner (2014), when the insert size is very short (i.e., less than the length of a single read), each paired read is a composite sequence starting with genomic insert sequence in 5′, whereas the downstream sequences contain oligonucleotides used for library preparation (i.e., reverse complement of concatenated primer + index + transposase sequences), followed by a short stretch of As, and next by apparently random sequence with low Phred (Ewing and Green, 1998) quality scores *Q* (see Figure 1A). In order to trim off these exogenous oligonucleotide sequences, Turner (2014) compared the respective accuracy of cutadapt and AlienTrimmer. Yet, the two programs were run using as input to be trimmed off, only the transposase sequence, but not the other alien oligonucleotide sequences (see Figure 1A). With this incomplete setting, each trimming program performed quite differently. As cutadapt was set to perform 3′ trimming only, it detected the transposase sequence as well as downstream sequences. In order to provide more flexible functionality and simpler usage, AlienTrimmer's strategy is to detect the specified alien oligonucleotides within reads and perform trimming when the matched region is close enough to a read end. Therefore, when the adapter oligonucleotide (or transposase) sequence is far from the 3′ end because it is followed by unspecified index, primer or artefactual nucleotides, it is detected by AlienTrimmer but not trimmed off (see Figure 1A). As a consequence, AlienTrimmer did not yield accurate read trimming in the way Turner (2014) used it (i.e., without specifying index, primer and artefactual sequences, although they were identified).

**Figure 1:**
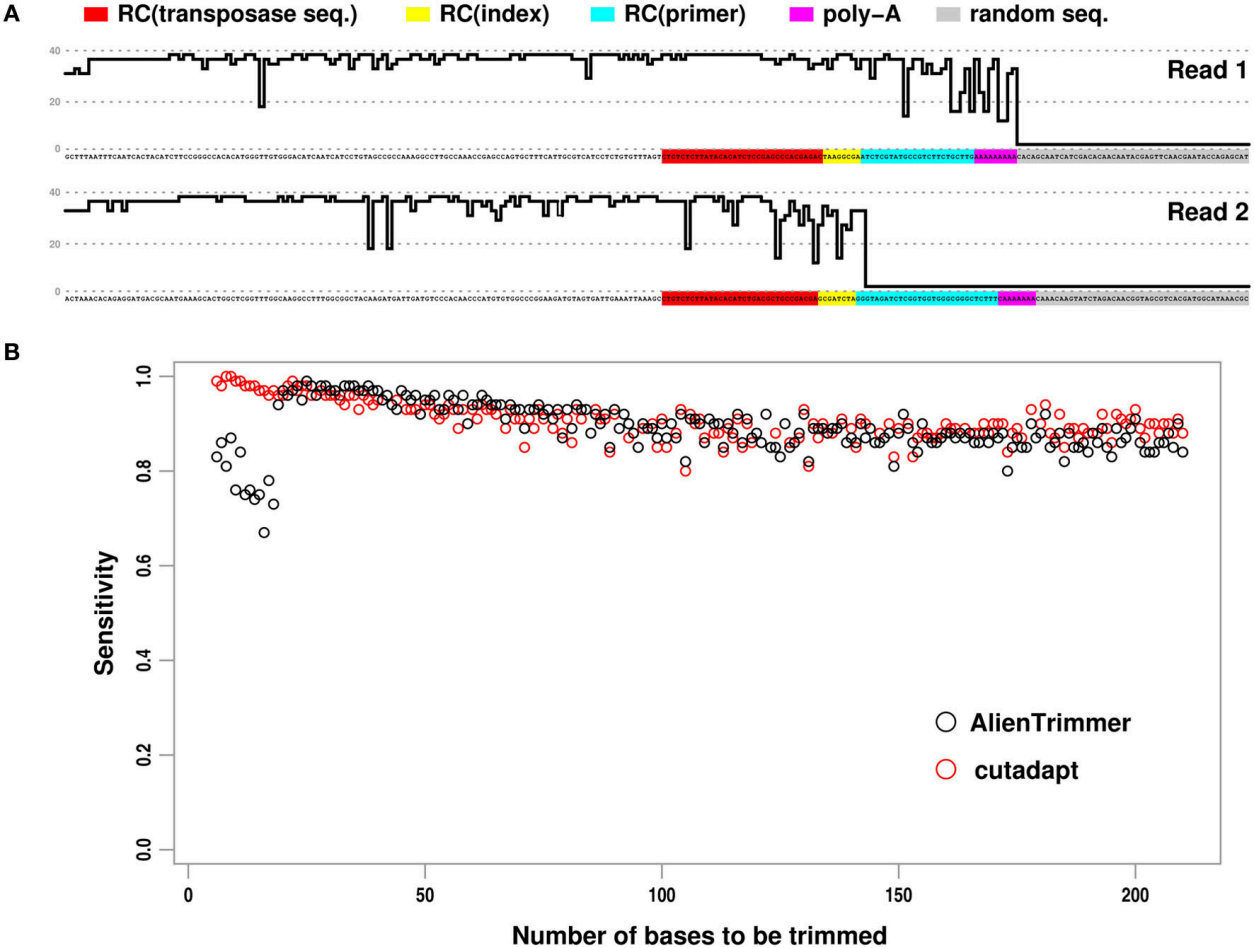
**Paired reads containing exogenous oligonucleotide sequences to be trimmed off, and performance results of two trimming programs. (A)** Example of short-insert paired-end read with small insert size. Phred quality score *Q* (up to 40) of each nucleotide is represented by skyline plot. For better reading, threshold *Q* = 0, 20, 40 are represented by dashed horizontal lines. RC, reverse complement. **(B)** Plot showing the estimated sensitivity of AlienTrimmer (black circles) and cutadapt (red circles) for each number of bases to be trimmed within short-insert paired-end reads.

Here, we compared cutadapt (version 1.3) and AlienTrimmer (version 0.3.2) on the original read data (Turner, 2014) by providing all oligonucleotide sequences to be trimmed off. Following Turner (2014) analyses, all paired reads with predicted insert size <250 bps were gathered, and every read pair of combined length <250 bps after quality trimming (cut-off *Q* = 20) was discarded, therefore leading to a subset of ~80,000 read pairs. The program cutadapt was launched with the two reverse complemented transposase sequences to perform 3′ end trimming, as it would run slower when specifying more oligonucleotide sequences, which is unnecessary (see above). As AlienTrimmer running times are not affected by the number of specified alien oligonucleotides to trim off, it was launched with all combination of exogenous bps (i.e., reverse complement of concatenated primer + index + transposase sequences for each of the 16 used indexes + poly-A). Quality-trimming option (cut-off *Q* = 20) was also set for both programs in order to trim off low quality random bps in 3′, sometimes occurring when the insert size is very small (see Figure 1A). Following Turner (2014) criteria, true positive and false negative results were defined as trimmed reads leaving less and more than five bps to be trimmed, respectively. Denoting *TP* and *FN* as the number of true positive and false negative results, respectively, the sensitivity *TP* / (*TP* + *FN*) was estimated for each number of bps to be trimmed (Figure 1B).

All read pairs with insert size <250 bps were processed by cutadapt and AlienTrimmer in ~17 and ~8 s, respectively, confirming that AlienTrimmer runs fast with multiple input alien oligonucleotides. Note that we ran the program AlienTrimmer compiled with gcj to native machine code to observe such fast running times: different Java virtual machines were tested to execute AlienTrimmer, each running from ~2 (Oracle JRE 7) to ~6 (gij version 4.8) times slower than the gcj-compiled version on these read data. Clearly, AlienTrimmer had high sensitivity. As expected, AlienTrimmer had moderate sensitivity only when the number of bps to be trimmed was small (i.e., less than the specified *k*-mer value within a single read, see Criscuolo and Brisse, 2013). Indeed, as AlienTrimmer was launched with default *k*-mer value (i.e., *k* = 10), moderate sensitivity was observed when the number of bps to be trimmed was less than 20. However, higher sensitivity values were observed by setting lower *k*-mer values (not shown). Of note, cutadapt plot in Figure 1B shows a decreasing trend that differs from the increasing one in Turner (2014). This could be explained by the fact that all paired reads with insert size <250 bps were analyzed here, whereas Turner (2014) considered selected subsets from each library.

In conclusion, AlienTrimmer has high sensitivity and speed in removing alien oligonuceotide sequences from short-insert paired-end reads. This work underlines that it is critical to specify all possible alien oligonucleotide sequences as input for AlienTrimmer (as allowed by the protocol presented by Turner, 2014) and to perform quality trimming upstream of alien sequence removal. As the speed of AlienTrimmer is not affected by the number of input oligonucleotide sequences, this feature is a strong advantage for the process of raw data from read archives, for which the oligonucleotide sequences used for library preparation may be undocumented.

## Conflict of interest statement

The authors declare that the research was conducted in the absence of any commercial or financial relationships that could be construed as a potential conflict of interest.

## References

[B1a] Bolger A. M. Lohsel M. Usadel B. (2014). Trimmomatic: a flexible trimmer for Illumina sequence data. Bioinformatics. 10.1093/bioinformatics/btu170 [Epub ahead of print]. PMC4103590 24695404

[B1] Criscuolo A. Brisse S. (2013). AlienTrimmer: a tool to quickly and accurately trim off multiple short contaminant sequences from high-throughput sequencing reads. Genomics 102, 500–506 10.1016/j.ygeno.2013.07.011 23912058

[B3a] Ewing B. Green P. (1998). Base-calling of automated sequencer traces using phred. II. Error probabilities. Genome Res. 8, 186–194 9521922

[B2] Marcel M. (2011). Cutadapt removes adapter sequences from high-throughput sequencing reads. EMBnet J. 17, 10–12 10.14806/ej.17.1.200

[B3] Turner F. S. (2014). Assessment of insert sizes and adapter content in FASTQ data from NexteraXT libraries. Front. Genet. 5:5 10.3389/fgene.2014.00005 24523726 PMC3906532

